# Australian health system restructuring – what problem is being solved?

**DOI:** 10.1186/1743-8462-1-6

**Published:** 2004-11-19

**Authors:** Judith M Dwyer

**Affiliations:** 1 Health Policy and Management Department, La Trobe University, Australia

## Abstract

**Background:**

In recent years, Australian state and territory governments have reviewed and restructured the health systems they lead and regulate. This paper examines the outcomes of the most recent official published reviews of systems and structures; identifies the common themes; and addresses two questions: what problems are being addressed? And how would we know if the changes were successful?

**Results:**

In all the broad, systemic reviews, the main health system problems identified were money, hospital utilisation and a weak primary health care system. The solutions are various, but there is a common trend towards centralisation of governance, often at state health authority level, and stronger accountability measures. Other common themes are hospital substitution (services to avoid the need for admission); calls for cooperation across the Commonwealth:state divide, or for its abolition; and the expected range of current efficiency and effectiveness measures (eg amalgamate pathology and support services) and ideas in good currency (eg call centres). The top-down nature of the public review process is noted, along with the political nature of the immediate catalysts for calling on a review.

**Conclusion:**

The long-standing tension between the pull to centralisation of authority and the need for innovation in care models is heightened by recent changes, which may be counterproductive in an era dominated by the burden of chronic disease. I argue that the current reforms will not succeed in achieving the stated goals unless they make a difference for people with chronic illness. And if this is correct, the most useful focus for evaluation of the success of the reforms may be their impact on the system's ability to develop and deliver better models of care for this growing group of patients.

## Background

In recent years, there has been a rolling (and sometimes repetitive) tide of structural change in the way state and territory governments organise to lead and/or provide health care within their jurisdictions, with every state and territory of Australia involved at least once in the last 10 years.

This paper examines the outcomes of the most recent official published reviews of systems and structures; identifies the common themes; and addresses two questions: what problems are being addressed? And how would we know if the changes were successful?

This analysis focuses on those reviews which are 'systemic' in the sense that they examine broadly the structure and performance of a state/territory health system, and/or address governance of the system. The NSW restructure has been included, although it differs from the others in the absence of an independent review process and in the related lack of detailed documentation of the rationale for change.

## Review of reviews

The most recent wave of systemic reviews in the Australian public health system saw New South Wales [1], South Australia [2], the Northern Territory [3], Western Australia [4] and the Australian Capital Territory [5] go in for restructuring. Victoria reviewed metropolitan health system governance [6], but pulled back from major structural change, having had a round of it in 2000 [7].

NSW has relinquished its status as an island of relative stability, which had been maintained since 1986 in spite of several reviews, penultimately by IPART [8]. In the aftermath of a scandal at MacArthur Health Service [9], the Minister announced the abolition of all Area Health Service boards, and is restructuring the health services into 8 'super-regions' with CEO's who report directly to the head of the Department [1]. Clinicians and the community will be represented on advisory structures, and a new agency will take over support functions.

Queensland stays with central control (virtually no boards of governance to dilute the Department's authority) while Tasmania is reviewing hospital services only [10], having restructured in 1991 (from 'atomised' to regionalised) and 1997 (from regionalised to centralised) [11].

## Results

The pattern of systemic reviews over the last 10 years is summarised in Table 1. One notable trend is that the decision to review is often no longer presented publicly as a matter solely for the health minister. The premier or a financial/regulatory arm of government (mostly in concert with the health minister) commissioned the most recent reviews in Western Australia, South Australia, and the Northern Territory. The NSW restructure arises from a different process than the others undergoing structural change, with a brief booklet announcing and explaining the decision [1], rather than extended public review processes with opportunities for community and health service provider input.

**Table 1:** Review Dateline

**Year**	**States**	**Year**	**States**
2004	WA, NSW, Tasmania (hospitals only)	2000	Victoria
2003	NSW, Victoria#, SA, NT	1996	Tasmania, Qland, ACT
2002	ACT,	1995	Victoria, SA
2001	WA		

Notes: #No major structural changes recommended – focus on governance

### The trend: centralisation of governance

In what has emerged as a strong centralising tendency, 6 of 8 jurisdictions have centralised governance authority for public sector health care agencies at the level of the state or territory health authority. Victoria and South Australia are mixed, with regionalised or 'networked' structures predominating in the capital city; and several different approaches to both regional and institutional governance elsewhere. As Somgen points out, Victoria and South Australia were the states most strongly influenced by the 1990's trend to privatisation, outsourcing and output-based funding [12], with less focus on structures and central planning.

Table 2 summarises the current arrangements by state, in order of population size, with the population shown in brackets in the left-hand column (M = million).

**Table 2:** Governance of public health care agencies in Australian states/territories

**State**	**Current Status**	**Recent changes**
**NSW 6.64M**	**Centralising by 1 January 2005; regionalised since 1986.**	**Moving from 17 Area Health Services with separate governance authority to 8 Area Health Services within Departmental governance.**
Victoria 4.87M	Rurals partly regionalised for many years; Melbourne 'networked' since 1995.	Melbourne networks restructured from 7 to 12 and names changed in 2000. Rural structures mix of regionalised and atomised.
**Q'land 3.71M**	**Centralised at state level since 1996 after 5 years of regionalisation.**	**Long history of centralisation with advisory hospital boards; Regional Health Authorities 1991–1996.**
**WA 1.93M**	**Centralised at state level in 2001/02.**	**Moved from 'atomised' in Perth to one board in 1997, governance centralised in 2001; state now centralised.**
SA 1.52M	Regionalised in rural areas since 1995; Adelaide partly regionalising.	Moved from atomised to regionalised, with 2 regional and 1 specialised health services in the capital as of July 2004.
**Tasmania 0.47M**	**Centralised at state level**	**Moved from atomised to regionalised in 1991; centralised at state level in 1997.**
**ACT 0.32M**	**Centralised (single city system)**	**Single board for Canberra established in 1996; abolished in 2002.**
**NT 0.2M**	**Centralised at territory Level**	**Never devolved. Some autonomous Aboriginal Health Services.**

The recent NSW decision means that there is now a strong predominance of governance at state health authority level, with two-thirds of the Australian population living in areas served by centralised health services.

The second notable trend is the virtual end of 'atomised' structures – stand-alone, single-service agencies (ie, hospitals, community health, or mental health services) in the public sector. There are of course exceptions (women's and children's hospitals may be the last ones standing in a few years), and the picture is different for non-government organisations (like district nursing) which are less amenable to restructuring.

### Common Themes

The most recent reviews in WA and SA are characterised by claims to radical change, based on both financial and health goals:

*'...incremental reform is no longer the pathway to a financially sustainable vision for WA. A fundamental re-prioritisation of the public health system is needed, and should be carried out over the next decade in a systematic and integrated way' *[4], *p v*).

*'The people of South Australia have a decision to make on what type of health system they need now and for the future generation...there needs to be a significant shift from a system focused on illness to a health system reoriented towards health promotion, illness prevention and early intervention' *[2]*p xiii*.

Western Australia is taking on the tertiary hospitals, and reducing the number of tertiary sites from 5 to 2 (with the women's and children's hospital group to be collocated but organisationally separate). All state-run health services are to report through three metropolitan regions (north, south and Women's and Children's) and one rural region, with the CEO's reporting directly to the Department – there are to be no boards of governance. South Australia has succeeded in amalgamating most of Adelaide's hospital and community health boards to form 2 regional health services and 1 child, youth and women's health service (incorporating the women's and children's hospital). This is a notable achievement for a minority government, after at least five separate attempts in the last 20 years to rebalance power and responsibility had largely failed [13-17].

Not all of the systemic reviews claim to set a bold new vision, but there are strong common themes. The reports tell a familiar story of the need to bring increases in state health spending to sustainable levels, set against the trend of increasing costs due to increasing incidence of chronic disease, and more technologies for intervention, in an ageing population. They all focus on the need to improve quality and safety for patients.

The reviews also find that the health system is too fragmented to meet the needs of patients with long-term complex conditions well. This is seen to be partly because the system was designed for acute illness, with the current funding mechanisms also designed primarily on the pattern of acute interventions. The reviews call for better integration of services, so that navigating the system is easier for patients, their carers and care providers.

The reviews consistently argue that in order to achieve this, the primary care system needs to be more effective in managing or coordinating patients' needs for several different kinds of services when and where they are needed. The inevitable corollary is that inpatient care and hospitals have to become less central in the organisation and funding of the system. What can be done elsewhere should be; and the primary care level must have more of the action and more of the pulling power.

In turn, this will require different facilities for different modes of service delivery; different funding allocations and methods of allocation; and a solution to the atomisation of primary care caused by the Commonwealth/state split and the current model of fee-for-service medicine. The need for changes in the private sector is noted in the reviews, but proposals are not developed, because the states have such a limited role here.

Much is also made of the need for providers to be more accountable to government and the community, and/or better governed and managed. While the reports mostly call for less micro-managing from the head offices of health authorities, and a better separation between the roles of central policy-makers and peripheral service providers (or regional CEO's), there is also a countervailing tendency to recommend tighter engagement and control. For example, the Kibble review of governance in Victoria (2003) notes confusion about relative roles and responsibilities and calls for the Department of Human Services to reduce 'attention to the day-to-day operations of Health Services and monitoring of detailed activities' (p 27) but later recommends 'a standardised reporting template' for internal reports to boards across the system (p 35), along with stronger accountability for the CEOs to the Secretary of the Department. More public reporting of service outcomes and activity levels is a related common theme, intended to inform the public and to underpin attention to safety and quality.

The final major common theme in the reviews is the inclusion of an opportunistic range of technical efficiency and effectiveness measures, picking up ideas in good currency or known productivity opportunities. For example, almost everyone recommends a call centre; a web-based method of sharing innovations; amalgamation of support services where relevant; and improvements in the effectiveness of information systems and the use of information.

There are two other commonalities worth noting. Firstly, it is a fact of organisational and political life that official reviews are a top-down affair, commissioned by one level of the system to examine a lower level. Thus it is not surprising that there are no published official reviews of the roles and responsibilities of the Commonwealth health authority in the last twenty years. When the published reviews do address the roles and responsibilities of state health authorities, it is either because they *are *the providers (NT, WA, ACT) or because intended changes to the service provider level of the system require changes to the roles of central health authorities.

This is an important limitation in the current environment, when some of the key barriers to improving the effectiveness of health care delivery lie in the system's seldom seen upper reaches. As the reviews note, the way that the Commonwealth/state split of responsibility for health is enacted and managed is probably the single most significant problem in health system design. But it is not the only one. I refer not so much to important policy settings (like funding allocation models and public health priorities) which are studied and articulated in publicly-available documents, but rather to the influence of administrative decisions (like who gets special grants and who doesn't) and the effectiveness of relationships with health care provider organisations (as judged from the bottom up as well as the top down). The administrative actions of health authorities seem to go largely unexamined.

Secondly, while the underlying problems the reviews set out to address are all about money, hospital utilisation and a weak primary health care system, the immediate context is often the election of a new government (Victoria, SA, NT), the appointment of a new minister or health authority CEO (WA), media unrest about health in a state with a looming election (ACT) or scandal (NSW). This observation may simply be another way of saying that the health portfolio is highly politically sensitive as well as complex, and so risky that reference to independent expertise is seen as essential.

## Discussion

The main line of logic running through the recent reviews seems sound. The primary care system needs strengthening; what can be done outside hospitals should be; and a continuing focus on safety, performance and accountability is necessary.

The reports also make it clear that this is all about responding to the major challenge for the system: to improve its capacity to prevent, intervene early in, and manage chronic disease, the main driver of increased demand. Such a focus is clearly justified. Chronic disease is responsible for approximately 80% of the total burden of disease, with an estimated three million Australians suffering from one or more chronic illnesses [18]. About 40% of total health expenditure, or $12.6 billion, was spent on chronic illness in 1993/94, just less than half of it in hospitals [19]. The system must be able to deliver the kind of care needed by people with (or at risk of) chronic disease, including older people and Indigenous people, and thereby enhance the system's effectiveness and perhaps even reduce the slope of the increasing cost curve.

If this is the imperative, the trend away from atomised governance structures, and towards bringing multiple agencies which serve (at least some) common patients together, seems like the right direction. But there is another important requirement which may not be served by these moves – that is, a stronger focus on innovation in care models. While recommendations abound, we don't yet really know what will work best for the new pattern of illness – how do you best coordinate care around the needs of the chronicly ill?

Uncertainty about care models, and the institutional and policy arrangements needed to support them, can only be resolved through the continued development and testing of innovative approaches, on the ground in health care delivery, as happened most notably with the Coordinated Care Trials [20]. As many of the reviews argue, the engagement of clinicians is critical to this endeavour.

This reality implies that there is a secondary criterion by which the effectiveness of health system structural changes might be judged: do the changes enhance or inhibit the system's ability to innovate? The requirement for innovation and experimentation may not sit comfortably with government requirements for standardisation of known good practice. However in an area where best practice is not known, innovation is critical, and must be supported.

Unfortunately, the Commonwealth:state responsibility split, the one structural barrier most central to the systemic weakness of Australian primary care (and therefore most important for the capacity to develop and support new models of care for chronic diseases), is one that a state can't address, at least not alone. The Productivity Commission's recent call for an independent public review of the whole health system [21], focused on overlapping roles and responsibilities for funding, offers grounds to hope for movement in this otherwise intractable problem.

The other pessimistic sign is the trend to more direct control of health care provision by state governments, related no doubt to the twin problems of increasing demand (and therefore cost) and increasing disclosure of safety and quality problems, both of which can only politicise the system more. Research on innovation, in relation to quality and safety as well as other performance measures, indicates that micro-management from above is not helpful [22,23].

Local evidence to support this view is scant. While the effectiveness of the new arrangements during Queensland's brief period of devolved governance was judged harshly when it came time to re-centralise, some commentators suggest that this period also allowed Queensland to catch up to other states in areas like accreditation, casemix, IT and 'attention to performance management and outcomes' [24].

This may be a critical problem. While recognising that perspectives on this question are highly related to one's place in the structure, I would suggest that real innovation in public hospitals and health services is less likely to be driven by clinicians who are more tightly controlled, staff who have learnt to be risk-averse, or managers who are increasingly frightened of tomorrow's headlines, and whose planning horizon extends to next month's financial and activity data. This problem is only compounded while hospitals and community health services on the one hand, and GPs on the other, continue to work with so little in the way of common incentives.

## Conclusion

The recent reviews were established largely to address financial imperatives in an environment of upward pressure on demand for services, and accountability concerns (in relation to quality and safety, and general good governance), mostly in a highly political context. The reviewers rightly sought to take a longer-term strategic perspective. They attempted (with varying degrees of success) to focus on good system design and capacity to meet the broad and complex purposes of public health systems, recognizing the growing challenges the systems face.

Structural reform is hardly ever evaluated, other than when its weaknesses are articulated by those proposing the next round of changes, as part of the rationale for their efforts. There are many reasons for this failure, some of them political. One pertinent reason is that outcomes like containing the pressure of future growth in demand, or improving health outcomes for the population, cannot be judged within a realistic time frame.

However, in the current environment, with strong convergence in the themes addressed by a fairly comprehensive round of reviews of Australian health systems, an argument can be made for evaluation 'at the pointy end' of the changes. Given the challenges the reviews were intended to address, there are grounds to suggest that the current reforms will not succeed in achieving the stated (shorter- and longer-term) goals unless they make a difference for people with chronic illness. And if this is correct, the most useful immediate focus for evaluation of the success of the reforms may be their impact on the system's ability to develop and deliver better models of care for this growing group of patients.

## Methods

The 'data' for this project were the published reports of systemic reviews of the health systems, and related material published on departmental websites and in the professional and academic literature.

These sources were analysed to generate an understanding of the recommended governance authority structures; and the common themes emerging from the reasoning on which the recommendations were based. The themes underpinning the recommendations were then assessed in the light of the overarching goals of reform.

## Competing Interests

I was a member of the South Australian 'Generational Health Review' Steering Committee, chaired its Governance and Funding Task Force and continue to consult to the SA Department of Human Services. I worked for twenty years in health care agencies, and only one in a central health authority.
